# Can the Pain Attitudes and Beliefs Scales be adapted for use in the context of osteoarthritis with general practitioners and physiotherapists?

**DOI:** 10.1186/s41927-020-0116-1

**Published:** 2020-04-10

**Authors:** Daniel W. O’Brien, Sandra Bassett, Valerie Wright-St Clair, Richard J. Siegert

**Affiliations:** 1 Physiotherapy Department, School of Clinical Sciences, Auckland University of Technology, North Shore Campus, Akoranga Drive, Northcote, Auckland, 0627 New Zealand; 2 Occupational Therapy Department, Auckland University of Technology, Auckland, New Zealand; 3 Psychology Department, Auckland University of Technology, Auckland, New Zealand

**Keywords:** Osteoarthritis, Health and illness beliefs, Quantitative measures

## Abstract

**Background:**

Conservative, first-line treatments (exercise, education and weight-loss if appropriate) for hip and knee joint osteoarthritis are underused despite the known benefits. Clinicians’ beliefs can affect the advice and education given to patients, in turn, this can influence the uptake of treatment. In New Zealand, most conservative OA management is prescribed by general practitioners (GPs; primary care physicians) and physiotherapists. Few questionnaires have been designed to measure GPs’ and physiotherapists’ osteoarthritis-related health, illness and treatment beliefs. This study aimed to identify if a questionnaire about low back pain beliefs, the Pain Attitudes and Beliefs Scale for Physiotherapists (PABS-PT), can be adapted to assess GP and physiotherapists’ beliefs about osteoarthritis.

**Methods:**

This study used a cross-sectional observational design. Data were collected anonymously from GPs and physiotherapists using an online survey. The survey included a study-specific demographic and occupational characteristics questionnaire and the PABS-PT questionnaire adapted for osteoarthritis. All data were analysed using descriptive statistics, and the PABS-PT data underwent principal factor analysis.

**Results:**

In total, 295 clinicians (87 GPs, 208 physiotherapists) participated in this study. The principal factor analysis identified two factors or subscales (categorised as biomedical and behavioural), with a Cronbach’s alpha of 0.84 and 0.44, respectively.

**Conclusions:**

The biomedical subscale of the PABS-PT appears appropriate for adaptation for use in the context of osteoarthritis, but the low internal consistency of the behavioural subscale suggests this subscale is not currently suitable. Future research should consider the inclusion of additional items to the behavioural subscale to improve internal consistency or look to develop a new, osteoarthritis-specific questionnaire.

**Trial registration:**

This trial was part of the primary author’s PhD, which began in 2012 and therefore this study was not registered.

## Background

Osteoarthritis is a chronic musculoskeletal condition that can affect a person’s physical, social and mental well-being [1]. Hip and knee joint osteoarthritis are currently ranked as the 11th highest contributor to disability worldwide [2]. International evidence-based practice guidelines recommend that osteoarthritis treatment options progress from conservative interventions (e.g. dietary changes and exercise) to invasive treatments (e.g. joint replacement surgery) [3–6]. Furthermore, treatment of osteoarthritis should be multi-faceted and reflect the different ways the disease can affect the individual, including physically, socially and psychologically [7–9]. A large component of the first-line conservative management of osteoarthritis (exercise, education and weight-loss if appropriate) occurs in primary care; in New Zealand, this care is typically provided by physiotherapists and general practitioners (GPs), known elsewhere as primary care physicians [10].

Despite first-line conservative treatments being considered the cornerstone of osteoarthritis management, research suggests conservative treatments for hip and knee joint osteoarthritis are underused [3, 6, 11–13]. Additionally, adherence to conservative treatments is poor and known to be affected by people’s health, illness and treatment beliefs [14–16]. Research among people with lower-limb osteoarthritis linked patients’ beliefs and attitudes to treatment choices and outcomes [17–20]. Patients often hold biomechanical or biomedical views of osteoarthritis, driven by a belief that the disease is primarily caused by joint wear and tear [21, 22]. This perception can lead to beliefs that conservative treatments lack efficacy and the only solution for osteoarthritis is a total joint replacement [23]. Clinicians’ beliefs and attitudes are known to affect the advice and education they offer their patients, and researchers have suggested that clinicians with biomedical or biomechanical beliefs about osteoarthritis may transfer these beliefs to their patients, thus affecting their treatment choices [6, 24, 25]. However, in contrast to patients’ beliefs about osteoarthritis, less is known about clinicians’ beliefs about the disease and its treatment, how clinicians’ beliefs may affect clinical practice, or how to best measure these beliefs [26–29].

Health, illness and treatment belief models are commonly used in research to explore, explain and understand patients’ behaviours [30–32]. Although clinician-focused health, illness and treatment belief models are not conventionally used in research, they are primarily used as frameworks to guide clinical practice in Western medicine [33]. These frameworks are traditionally medically driven, with the most recognised being the biomedical and biopsychosocial models [34]. Osteoarthritis treatment guidelines state that clinicians should use a biopsychosocial approach when treating people with osteoarthritis [9], but the extent to which clinicians’ beliefs about osteoarthritis match a biopsychosocial view of the disease remains unknown.

Few studies have explicitly explored clinicians’ osteoarthritis health, illness and treatment beliefs using a valid and reliable quantitative measure underpinned by a health beliefs model. Most existing measures or questionnaires about clinicians’ beliefs primarily focus on beliefs about low back pain (LBP), few questionnaires have been developed for use in the context of osteoarthritis [29, 35–37]. The most rigorously tested of these questionnaires is the Pain Attitudes and Beliefs Scale for Physiotherapists (PABS-PT). The questionnaire is underpinned by the biomedical and biopsychosocial models [35]. The PABS-PT has previously performed well in terms of internal consistency, reliability and validity testing, and the scale has the potential to be adapted for application in the osteoarthritis context [35, 37–40]. Recently, the questionnaire has been used to measure clinicians’ beliefs about osteoarthritis [29], but it is unclear if the questionnaire is appropriate in this context. This study aimed to determine if the PABS-PT is suitable for adaption for use in the context of osteoarthritis with general practitioners and physiotherapists.

## Methods

### Study design

This cross-sectional, observational study was part of a larger mixed-methods project [41]. Data were collected via online questionnaires. This paper presents the data collected with the PABS-PT [41].

### Participants

Clinicians were eligible to participate if they were registered and practising in New Zealand as either a physiotherapist or GP, had treated a patient with hip and/or knee osteoarthritis in the past 6 months, were living in New Zealand at the time of data collection and had sufficient English language skills to complete the survey.

### Measures/questionnaires

The survey comprised two sections: 1) demographic and occupational characteristics and 2) beliefs about hip and/or knee joint osteoarthritis (adapted PABS-PT). Demographic and occupational characteristics data included participants’ sex, age, duration of practice and geographical location of practice.

The PABS-PT was originally designed to collect physiotherapists’ beliefs about the treatment of LBP [35]. Since then, the measure has been adapted for use by both GPs and physiotherapists [37], including to measure beliefs about neck pain [40]. The present study used the original version of the questionnaire, which comprises 20 items that are scored on a six-point Likert scale (*totally disagree* to *totally agree*) [35]. The questionnaire has a two-factor structure. One factor is labelled biomedical (14 items) and the other behavioural (6 items). For this study, the questionnaire was adapted so that any reference to LBP was replaced with ‘osteoarthritis’. Examples of items are: ‘*Pain caused by osteoarthritis indicates the presence of organic injury’* and *‘The cause of osteoarthritic pain is unknown’.*

### Procedure

Before the survey was administered, all questions were tested for face validity and readability. Three researchers with experience in osteoarthritis research and survey design read the questionnaire and provided feedback about survey length, appropriateness for the New Zealand context and readability. The fully anonymised survey was advertised through several channels: physiotherapy continuing education courses; the Physiotherapy New Zealand Conference; Physiotherapy New Zealand and The Royal New Zealand College of General Practitioners e-newsletters; and the local primary healthcare organisation. Data were collected between 1 September and 1 December 2016 via SurveyMonkey (https://www.surveymonkey.com). Participants were required to read the online participant information sheet and respond to the items in the questionnaire. No identifying information was collected, and participants could not be identified or traced.

### Data analysis

All data were analysed using SPSS version 24.0 (IBM, USA), with the alpha level set at *p* < 0.05. Missing data were limited by the use of the online platform because participants were directed by automatic prompts to complete any missed item or question. Only complete data sets were analysed. It was not possible to calculate a total return rate for the survey as participants completed the study online, and it was unknown how many potential participants saw the study advertisement but chose not to participate.

### Demographic and occupational characteristics

All data describing demographic and professional characteristics were categorical. For each category, the total number of scores was described using descriptive statistics. Data from GPs and physiotherapists were presented together and separately to allow comparison between the two professions. Categories that represented a small number of participants were collapsed into a single category, called ‘Other’. Group equivalency between the two professions for demographic and occupational characteristics data were assessed with chi-square tests [42]. The Yates correction for continuity was reported where data were represented as a two-by-two assessment [42].

### Factor structure of the adapted PABS-PT

Means and standard deviations were calculated to show the response distribution of the data for each item in the Adapted PABS-PT. The correlation matrix was then calculated and screened to ensure the presence of correlations of 0.3 or greater, and to determine if the sample was suitable for principal component analysis. The data were subjected to the Kaiser-Meyer-Olkin measure of sampling adequacy and Bartlett’s test of sphericity, and principal component extraction was performed. On the basis of the Scree test, which was consistent with the number of factors reported by Ostelo et al. [35], the criteria were limited to a two-factor solution, and the eigenvalues for each factor were plotted. Varimax rotation was applied because it typically produces clear and interpretable solutions, and it would allow ready comparison with the findings of Ostelo et al. [35]. For clarity, factor loadings of 0.45 or lower were concealed. Next, each subscale (factor) was named, and its internal consistency examined by calculating the Cronbach’s alpha. Where appropriate, the names of subscales matched those proposed by Ostelo et al. [35]. The subscale was described as having acceptable internal consistency when the included items made conceptual sense and had a Cronbach’s alpha ≥0.7. Finally, group mean scores and standard deviations were calculated for each subscale.

### Ethical considerations

This study was granted ethical consent by the relevant Institutional Ethics Committee on 11 August 2016 (AUTEC: 16/284). There was no mechanism of identifying people who participated in the study and the participant information explained that by submitting the questionnaire electronically, they were consenting for the data to be used for the study.

## Results

### Demographic and occupational characteristics

In total, 295 clinicians participated in this study and completed the demographic and occupational characteristics section of the survey (Table 1). The dropout rate from those who started the survey was 7.8%. Approximately 70% of participants were physiotherapists. More females (62.4%) than males completed the survey, irrespective of profession. The duration of practice ranged from less than 5 years to over 20 years in both professional groups. Participants from both professions came from a range of geographical and employment settings. GPs saw significantly more people with hip and/or knee osteoarthritis than physiotherapists.

**Table 1:** Participants’ demographic and occupational characteristics

Characteristic	Total n (%)	GPs n (%)	Physiotherapy n (%)
Participants	295 (100)	87 (29.5)	208 (70.5)
Sex			
Male	111 (37.6)	39 (44.8)	72 (34.6)
Female	184 (62.4)	48 (55.2)	136 (65.4)
Duration in practice, years			
< 5	60 (20.3)	9 (10.3)	51 (24.5)
6–10	63 (21.4)	20 (23.1)	43 (20.7)
11–15	38 (12.9)	11 (12.6)	27 (13.0)
16–20	38 (12.9)	8 (9.2)	30 (14.4)
> 20	96 (32.5)	39 (44.8)	57 (27.4)
Location of clinical practice			
City	197 (66.7)	54 (62.1)	143 (68.8)
Town	63 (21.4)	17 (19.5)	46 (22.1)
Rural	35 (11.9)	16 (18.4)	19 (9.1)
Employment setting			
Public (i.e. DHB or hospital)	51 (17.3)	4 (4.6)	47 (22.7)
Private (i.e. private practice)	218 (73.9)	74 (85.1)	144 (69.2)
Both	15 (5.1)	6 (6.9)	9 (4.3)
Other ^a^	11 (3.7)	3 (3.4)	8 (3.8)
Frequency of treating patients with hip and/or knee osteoarthritis			
1 or more patients per day	81 (27.5)	35 (40.2)	46 (22.1)
1–3 patients per week	121 (41.0)	43 (49.4)	78 (37.5)
1–3 patients per month	66 (22.4)	8 (9.3)	58 (27.9)
1–3 patients in the past 6 months	27 (9.1)	1 (1.1)	26 (12.5)

*DHB* District Health Board

^a^Other employment settings were: aged care (*n* = 1), community care service (*n* = 2), hospice care (*n* = 1), Māori health trust (*n* = 2), occupational health service (*n* = 1,) primary health organisation (*n* = 2) and university clinic (*n* = 2)

### Factor structure

In total, 285 complete individual’s data sets were included in the factor analysis. Table 2 shows the individual item group mean and standard deviation scores. The mean score for most questionnaire items was between 2 and 5. The correlation matrix identified correlations of 0.3 or greater, which indicated the data were suitable for principal component analysis. The Kaiser-Meyer-Olkin measure of sampling adequacy was 0.836, which was above the recommended value of 0.6, and Bartlett’s test of sphericity was significant (χ^2^ (190) = 1173.26, *p* < 0.0001). The analysis of the Scree test supported two-components and the principal component analysis supported the previously identified two-factor solution [35], with the two rotated factors explaining 32.29% of the total variance. The item loading on the first unrotated principal component and the rotated individual item loadings are shown in Table 2. Two items did not load onto either factor (*‘There is no effective treatment to eliminate pain caused by osteoarthritis’* and *‘A patient suffering from severe pain caused by osteoarthritis will benefit from physical exercise’*). The Cronbach’s alpha for the entire scale was acceptable (0.75). The subscale mean scores, Cronbach’s alpha scores, eigenvalues and percentage variance explained are shown in Table 3.

**Table 2:** Means, standard deviations and rotated two-factor analysis of the Adapted Pain and Attitudes Beliefs Scale – Physiotherapy

PABS-PT item			Factor^b^	Factor^b^
	Mean (SD)	Item Loading	1	2
If patients complain of pain during exercise, I worry that damage is being caused.	2.42 (1.05)	0.65	**0.65**	
If therapy does not result in a reduction in pain caused by osteoarthritis, there is a high risk of severe restrictions in the long term.	3.40 (1.22)	0.58	**0.64**	
Patients with pain caused by osteoarthritis should preferably practice only pain-free movements.	2.45 (1.07)	0.60	**0.62**	
Pain is a nociceptive stimulus, indicating tissue damage.	2.65 (1.15)	0.63	**0.60**	
The best advice for pain caused by osteoarthritis is: ‘Take care’ and ‘Make no unnecessary movements’.	1.53 (0.77)	0.59	**0.59**	
Patients who have suffered osteoarthritic pain should avoid activities that stress the joint.	2.88 (1.22)	0.60	**0.59**	
Pain reduction is a precondition for the restoration of normal functioning.	3.70 (1.22)	0.59	**0.58**	
The severity of tissue damage determines the level of pain.	2.14 (1.08)	0.59	**0.52**	
Not enough effort is made to find the underlying organic causes of pain caused by osteoarthritis.	3.18 (1.07)	0.48	**0.51**	
Pain caused by osteoarthritis indicates the presence of organic injury.	3.06 (1.09)	0.54	**0.51**	
Reduction of daily physical exertion is a significant factor in treating pain caused by osteoarthritis.	2.43 (1.20)	0.51	**0.51**	
If osteoarthritic pain increases in severity, I immediately adjust the intensity of my treatment accordingly.	3.89 (1.07)	0.50	**0.50**	
It is the task of the physiotherapist or GP to remove the cause of osteoarthritic pain.	2.29 (1.16)	0.47	**0.47**	
Increased pain indicates new tissue damage or the spread of existing damage.	2.85 (1.09)	0.55	**0.47**	
There is no effective treatment to eliminate pain caused by osteoarthritis.^a^	2.40 (1.20)	0.24		
Psychological stress can contribute to pain caused by osteoarthritis even in the absence of significant tissue damage.	5.02 (0.87)	−0.27		**0.62**
Functional limitations associated with pain caused by osteoarthritis are the result of psychosocial factors.	3.46 (1.04)	0.18		**0.59**
Knowledge of the tissue damage is not necessary for effective therapy.	4.05 (1.29)	−0.24		**0.55**
The cause of osteoarthritic pain is unknown.	2.86 (1.08)	0.06		**0.54**
A patient suffering from severe pain caused by osteoarthritis will benefit from physical exercise.^a^	5.07 (0.97)	−0.20		

*GP* general practitioner, *PABS-PT* Pain and Attitudes Beliefs Scale – Physiotherapy, *SD* standard deviation

^a^indicates items that did not load on to any factor with a score a score greater than .45. Only item scores greater than .45 are included on the table. Scores shown in bold indicate those that comprised the factor. ^b^Factor names: 1 = Biomedical, 2 = Behavioural

**Table 3:** Means, alphas, eigenvalues and variance explained percentages for the Adapted Pain and Attitudes Beliefs Scale – Physiotherapy

Subscale (factor) number	Subscale title	Subscale mean score (SD)	Cronbach’s alpha	Eigenvalues	Percentage of variance explained
1	Biomedical	2.78 (0.63)	0.84	4.74	23.71
2	Behavioural	3.85 (0.66)	0.44	1.72	8.58

*SD* standard deviation

## Discussion

This study aimed to identify if the PABS-PT could be adapted for use in the context of OA. The low internal consistency of the behavioural (biopsychosocial) sub-scale of the PABS-PT indicates that, in its current form, it is not suitable for use in the context of osteoarthritis.

The low internal consistency of the behavioural subscale raised questions about the usefulness of the subscale and indicated the measure was unable to assess the construct reliably. While previous reports of the internal consistency (Cronbach’s alpha) of the biomedical subscale have been acceptable (ranging from 0.75–0.84), the behavioural subscale is less consistent, ranging from 0.54–0.73 in the context of LBP [35, 40]. Of interest, recently, Briggs et al. [29] used the PABS in their study of clinicians’ osteoarthritis beliefs but only used the biomedical subscale. The low internal consistency of the behavioural subscale may be attributable to three factors. First, the limited ability of the subscale items to fully explain the complexity of the construct. The subscale has been amended and modified by several authors to resolve this problem, but this remains an issue [37, 39, 43]. This issue may be compounded by inconsistent interpretation of the behavioural items. Ip et al. [44] indicated that such problems could relate to differences in the belief anchors that link a belief to either the biomedical or biopsychosocial belief systems. Those authors explored health and illness beliefs among people with diabetes and found that biomedically-located anchors were reported more consistently than other anchors [44].

Second, continued issues with the internal consistency may relate to the complex nature of the behavioural (biopsychosocial) construct. When first proposed, the biopsychosocial model of health comprised four components that were equally important for a person’s well-being: biological, psychological, social and cultural [34]. The PABS-PT places biomedical beliefs in one subscale and behavioural beliefs (comprising psychological, social and cultural statements) in another subscale [35, 37]. Researchers argue that these three behavioural belief components represent very different aspects of a person’s well-being, and therefore cannot necessarily be grouped as a single construct [45]. Furthermore, the PABS-PT behavioural subscale typically comprises a small number of items [40]. This limited number of items cannot convincingly explore such diverse and complex notions of well-being.

Third, the biomedical and biopsychosocial models cannot be conceptualised as being independent. The biopsychosocial model of healthcare delivery was developed as an extension of the biomedical model, not as an independent model [34]. Therefore, attempting to create a scale that places beliefs into one of two categories (biomedical or biopsychosocial) may be conceptually flawed because the two categories are interdependent. The biomedical approach to healthcare is an important part of the biopsychosocial model. Consequently, attempting to differentiate biopsychosocial beliefs from biomedical beliefs may not be possible. Recently, Duncan et al. [45] used concept mapping to explore clinicians’ conceptualisation of the biopsychosocial approach in the context of musculoskeletal care. Those authors proposed a complex interpretation of the biopsychosocial model that included six primary domains: bio-clinical, therapeutic relationship, individual patient aspects, emotions, social and work [45]. Other researchers have explored the complexity of how clinicians conceived their approach to clinical practice [46]. Thomson et al. [46] proposed a more intricate conceptualisation of clinical practice than suggested by the biopsychosocial approach. They argued that clinicians’ conceptions of clinical practice are influenced by multiple factors, including their educational experience, view of health and disease, the epistemology of practice knowledge in which they practice, the theory-practice relationship and their perceived therapeutic role [46]. Moreover, clinical practice can be further affected by the therapeutic relationship, and whether the clinician employs a patient- or practitioner-centred approach to care [46].

The present study had three strengths. First, the sample size allowed for appropriate statistical analysis of the Adapted PABS-PT. Second, the demographic and occupational characteristics indicated that participants were representative of the wider population of GPs and physiotherapists in New Zealand. Third, the online administration of the survey enabled wide dissemination. However, this study also had two main limitations. First, the high survey dropout rate (7.8%) may reflect survey fatigue and indicate that the survey was too long for some participants. Second, the use of an online data collection method after broadly advertising the survey meant that a return rate could not be reported.

## Conclusions

This study suggests that the PABS-PT in its current form is not suitable for adaptation for use with GPs and physiotherapists in the context of osteoarthritis. Future research could consider including additional items to the behavioural subscale, as an attempt to improve the internal consistency of the subscale or consider the development of a new questionnaire to assess clinicians’ osteoarthritis beliefs.

## Data Availability

The dataset used in the present study are available from the corresponding author on reasonable request.

## Abbreviations

GP: General practitioner (primary care physician)
LBP: Low back pain
PABS-PT: Pain Attitudes and Beliefs Scale – Physical Therapy
TOA: Treatment beliefs in OsteoArthritis questionnaire
